# Transfemoral Transcatheter Aortic Valve Implantation (TF‐TAVI) for Patients With Left Ventricular Assist Device (LVAD) and Aortic Regurgitation

**DOI:** 10.1002/ccd.70213

**Published:** 2025-09-25

**Authors:** Daniel Tai‐Leung Chan, Ho‐On Alston Conrad Chiu, Ka‐Lam Wong, Ka‐Chun Un, Kwong‐Yue Eric Chan, Shu‐Yue Sze, Chor‐Cheung Frankie Tam, On‐Yat Wong, Gilbert H. L. Tang, Simon Cheung‐Chi Lam

**Affiliations:** ^1^ Department of Cardiothoracic Surgery Queen Mary Hospital Hong Kong China; ^2^ Department of Medicine, Li Ka Shing Faculty of Medicine, Cardiology Division The University of Hong Kong Hong Kong China; ^3^ Cardiology Division, Department of Medicine Queen Mary Hospital Hong Kong China; ^4^ Cardiac Medical Unit Grantham Hospital Hong Kong China; ^5^ Department of Cardiothoracic Anaesthesia Queen Mary Hospital Hong Kong China; ^6^ Department of Cardiovascular Surgery Mount Sinai Health System New York USA

**Keywords:** aortic regurgitation, left ventricular assist device, transcatheter aortic valve implantation

## Abstract

Aortic regurgitation (AR) develops in up to 25%−30% of patients with left ventricular assist device (LVAD). Treatment remains challenging since surgery confers significant peri‐operative risk and the lack of valvular calcification renders transfemoral transcatheter aortic valve implantation (TF‐TAVI) with non‐dedicated devices technically challenging. We present a case series wherein a TF J‐Valve system, a dedicated transcatheter heart valve for pure AR, emerges as an alternative treatment for AR in patients with an LVAD. We demonstrate successful deployment even in the presence of challenging anatomy including aortic root thrombus. Technical considerations when performing TF‐TAVI in LVAD patients are discussed.

**Abbreviations**
ACT: activated clotting time
Ao: aorta
AR: aortic regurgitation
AV: aortic valve
BCA: brachiocephalic artery
CCA: common carotid artery
CRTD: cardiac resynchronization therapy device
CT: computed tomography
HM III: HeartMate III
ICU: intensive care unit
INR: International normalized ratio
LA: left atrium
LCA: left coronary artery
LCC: left coronary cusp
LV: left ventricle
LVAD: left ventricular assist device
LVOT: left ventricular outflow tract
NCC: non‐coronary cusp
NYHA: New York Heart Association
RA: right atrium
RCC: right coronary cusp
SAVR: surgical Aortic Valve Replacements
STJ: sino‐tubular junction
TEE: transesophageal echocardiography
TF‐TAVI: transfemoral transcatheter aortic valve implantation
THV: transcatheter heart valve
VC: vena contracta
VCA: vena contracta area

## 1 Introduction

Aortic regurgitation (AR) complicating left ventricular assist device (LVAD) implantation is present in up to 25%−30% of LVAD recipients [1] with consequent ineffective pump output due to recirculation of antegrade blood flow. Studies have demonstrated that development of AR in LVAD recipients is associated with poorer prognosis [2]. Treatment of AR in LVAD recipients remains challenging since surgery confers significant peri‐operative risk, while the lack of valvular calcification renders transfemoral transcatheter aortic valve implantation (TF‐TAVI) technically challenging. Furthermore, donor heart for transplantation may not be readily available. Previous case series mainly involved the use of the JenaValve (Edwards Lifesciences, USA) or non‐dedicated TAVI devices for pure AR [3]. We present a case series that demonstrates how the J‐Valve system (JC Medical, China), a dedicated transcatheter heart valve (THV) for pure AR, has emerged as an alternative treatment option for LVAD patients, particularly as a potential bridge to heart transplantation. A case of TF‐TAVI using a non‐dedicated device is included as a comparator, further highlighting the effectiveness and safety of dedicated THV for pure AR.

## 2 Case 1 (Non‐Dedicated Device)

**Figure 1:**
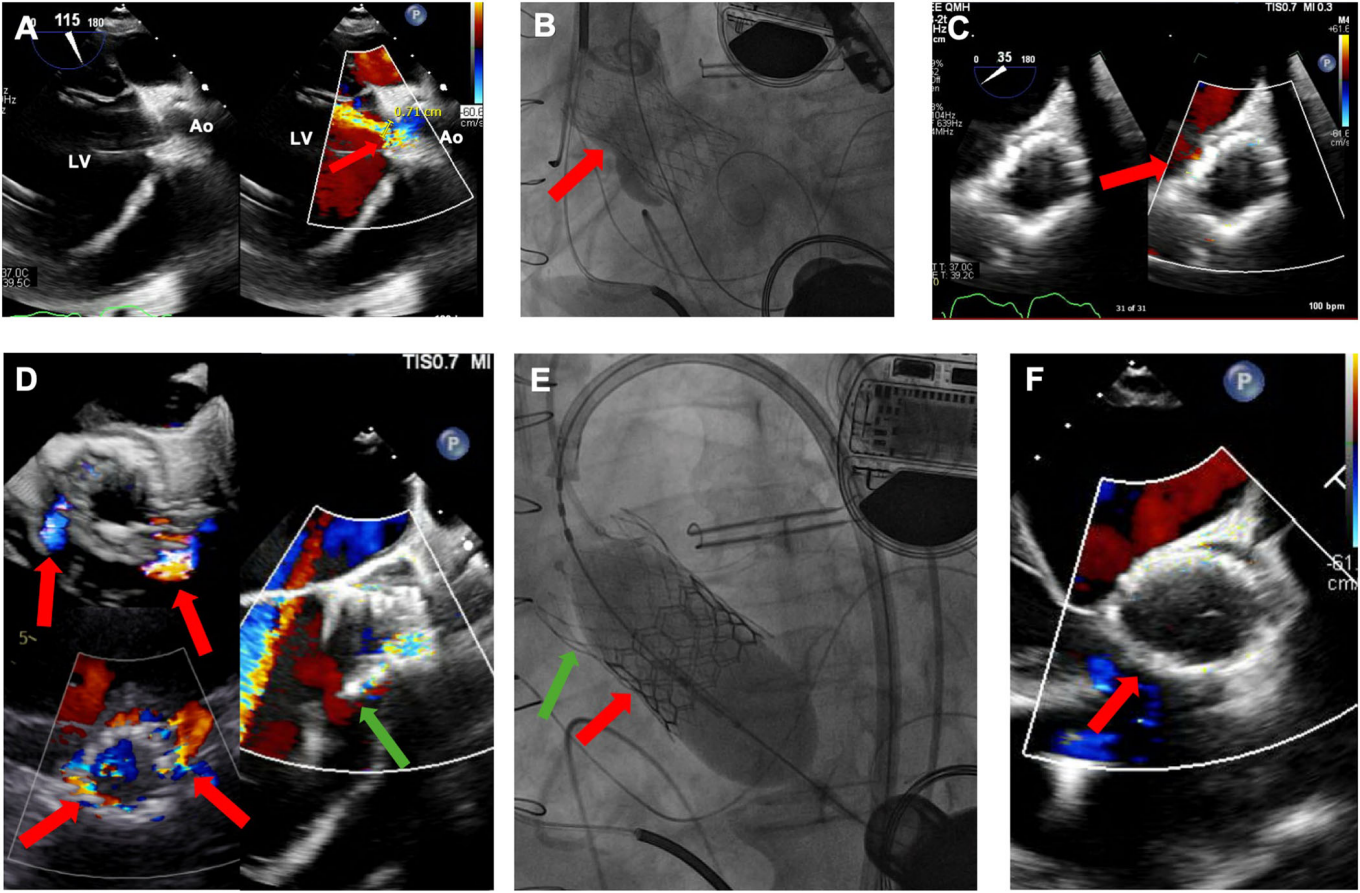
Patient 1. (A) Severe aortic regurgitation with vena contracta (VC) 0.7 cm [red arrow] echocardiography. (B) Successful Evolut R deployment confirmed on fluoroscopy [red—arrow Evolut R]. (C) Trivial AR following Evolut R deployment. (D) Severe paravalvular leak of Evolut R THV with downward migration of Evolut R THV toward left ventricular side [red arrow—paravalvular leak; green arrow—downward migration of Evolut R THV toward left ventricular side]. (E) Valve‐in‐valve TAVI with deployment of Edwards Sapien 3 THV within Evolut R THV [red arrow—Edwards Sapien 3; green arrow—Evolut R THV]. (F) Echocardiography showing trivial residual AR [red arrow—BEdwards Sapien3 within Evolut R]. [Color figure can be viewed at wileyonlinelibrary.com]

A 58‐year‐old patient who received a cardiac resynchronization therapy device (CRTD) for dilated cardiomyopathy developed progressive heart failure and required LVAD implantation with HeartWare in 2018. By 2021, he had developed symptomatic severe AR (vena contracta [VC] 0.7 cm) with NYHA class IV symptoms that required his repeated admission for diuresis. Despite optimizing medical treatment with losartan, eplerenone, and furosemide, the patient developed decompensating heart failure and required inotropic and intensive care unit (ICU) support in April 2021 (Figure 1A). Computed tomographic (CT) analysis revealed a bicuspid aortic valve (AV). Aortic annulus diameter, perimeter and area were 2.64 × 3.42 cm, 94.1 mm, and 704.6 mm^2^ respectively on CT (Table 1). Femoral access and coronary heights were favourable for transfemoral TAVI. In view of his acute decompensated heart failure and need for inotropic support, the patient was deemed high risk for open surgical valve replacement (SAVR). Donor heart for transplantation was also not available. Since a dedicated TF‐TAVI system for pure AR was not yet available at our unit, the heart team consensus was to perform TF‐TAVI with Evolut R #34 mm under general anaesthesia in April 2021. Peri‐procedurally, the patient was on aspirin and warfarin without interruption, with international normalised ratio (INR) maintained at 2.0−3.0 throughout. After crossing the AV with a straight‐tip emerald wire via an AL1 catheter, the Evolut R THV was delivered across the AV on a SAFARI large wire (Boston Scientific, USA). After reducing LVAD speed flow from 2580 to 1800 rpm, the Evolut R THV was successfully deployed (Figure 1B). LVAD flow speed was subsequently restored to 2400 rpm. Following THV deployment, sequential balloon dilatation using a TRUE balloon 26 mm × 4.5 cm and 28 mm × 4.75 cm was performed under rapid pacing (at 180 bpm) to optimize THV expansion. Activated clotting time (ACT) was maintained > 300 s throughout the procedure with parenteral heparin. The patient showed initial clinical improvement with NYHA function class I and trivial AR on echocardiography on discharge (Figure 1C). Two weeks following the index procedure, the patient developed symptoms of recurrent fluid overload and required ICU admission for diuresis and inotropic support. Re‐assessment echocardiography revealed a severe paravalvular leak at 1−3 o'clock and 6−9 o'clock respectively, along with downward migration of the THV (Figure 1D). The heart team consensus was to perform emergency valve‐in‐valve TF‐TAVI using Edwards SAPIEN 3 #29 mm THV. As in the index procedure, warfarin was continued throughout the procedure with INR maintained at 2.9. After crossing the AV with a 5 Fr Pigtail, a SAFARI small wire (Boston Scientific, USA) was introduced into the left ventricle. After introducing the commander delivery system, the Edwards SAPIEN 3 #29 mm THV (nominal volume + 4 mL) was advanced across the former Evolut R THV. After reducing LVAD speed flow from 2400 to 1800 rpm, the Edwards SAPIEN 3 THV was successfully deployed under rapid pacing at 180 bpm (Figure 1E). LVAD flow speed was subsequently restored to 2460 rpm. Post‐dilatation using the same delivery balloon under rapid pacing (at 180 bpm) was performed to optimize THV expansion. ACT was maintained at > 300 s with parenteral heparin throughout the procedure. The patient was inotrope‐free the following day, and remained at NYHA function class I on discharge. He continued warfarin with INR maintained at 2.0−3.0 for LVAD. At 1‐ and 2‐year follow‐up, reassessment echocardiography showed only trivial residual AR, with no evidence of AV or aortic root thrombus (Figure 1F). NT‐proBNP level improved from 9982 to 3118 ng/L. He remained at NYHA function class I at 2‐year follow‐up with no further hospitalizations for heart failure.

**Table 1:** Patient characteristics.

	Patient 1	Patient 2	Patient 3	Patient 4
Age	58	55	63	48
Gender	Male	Male	Male	Male
LVAD model	HeartWare	Heartmate III	Heartmate III	Heartmate III
Aortic valve morphology	Bicuspid	Tricuspid	Tricuspid	Tricuspid
AR type	Continuous	Continuous	Continuous	Continuous
AR VC	0.7 cm	0.6 cm	0.7 cm	0.8 cm
AR VCA	N/A	0.442 cm^2^	0.506 cm^2^	0.495 cm^2^
Aortic root clot	None	None	Yes (15 × 16 mm)	None
TEE aortic annulus diameter	N/A	23.2 × 28.4 mm	25.0 × 31.0 mm	18.0 × 24.0 mm
TEE aortic annulus area	N/A	523 mm^2^	629 mm^2^	368 mm^2^
TEE aortic annuus perimeter	N/A	83.2 mm	90.9 mm	69.4 mm
CT aortic annulus diameter	− Minimum: 26.4 mm − Maximum: 34.2 mm − Average: 30.3 mm − Area derived 30.0 mm − Perimeter derived 30.0 mm	− Minimum: 23.1 mm − Maximum: 28.3 mm − Average: 25.7 mm − Area derived: 25.8 mm − Perimeter derived: 26.0 mm	− Minimum: 24.6 mm − Maximum: 31.6 mm − Average: 28.1 mm − Area derived: 28.2 mm − Perimeter derived: 28.6 mm	− Minimum: 19.7 mm − Maximum: 22.7 mm − Average: 21.2 mm − Area derived: 21.3 mm − Perimeter derived: 22.0 mm
CT aortic annulus area	704.6 mm^2^	521.9 mm^2^	626.8 mm^2^	354.7 mm^2^
CT aortic annulus perimeter	94.1 mm	81.7 mm	90.0 mm	69.2 mm
CT aortic sinus diameter	26.3 × 41.0 mm	30.2 × 27.4 × 24.2 mm	35.9 × 31.8 × 33.4 mm	30.6 × 26.6 × 25.4 mm
CT STJ size	− Average: 30 mm	− Minimum: 24.7 mm − Maximum: 25.9 mm − Average: 25.3 mm	− Minimum: 31.0 mm − Maximum: 32.0 mm − Average: 31.7 mm	− Minimum: 24.1 mm − Maximum: 24.6 mm − Average: 24.3 mm
Pre‐TAVI NYHA class	IV	IV	IV	IV
Pre‐TAVI NT‐proBNP	9982 ng/L	1372 ng/L	4814 ng/L	15,955 ng/L
Baseline LVAD flow speed	1st THV: 2580 rpm 2nd THV: 2400 rpm	5000 rpm	4800 rpm	5000 rpm
Deployment LVAD flow speed	1st THV: 1800 pm 2nd THV: 1800 rpm	3000 rpm	3200 rpm	3000 rpm
Deployment temporary pacing	180 bpm	NIL	NIL	180 bpm
THV Valve size	1st THV: Evolut R #34 mm 2nd THV: Sapien3 #29 mm	J‐valve #28 mm	J‐valve #29 mm	J‐valve #25 mm
THV Oversizing	13.3%	7.7%	1.4%	13.6%
Post‐TAVI AR severity	Trivial	None	None	Trivial
Post‐TAVI NT‐proBNP	3118 ng/L	752 ng/L	1519 ng/L	4822 ng/L
Post‐J‐valve NYHA	I	I	I	I

Abbreviations: AR, Aortic regurgitation; CT, computed tomography; LVAD, left ventricular assist device; NYHA, New York Heart Association; STJ, sino‐tubular junction; TAVI, transcatheter aortic valve implantation; TEE, transesophageal echocardiography; VC, vena contracta; VCA, vena contracta area.

## 3 Case 2

**Figure 2:**
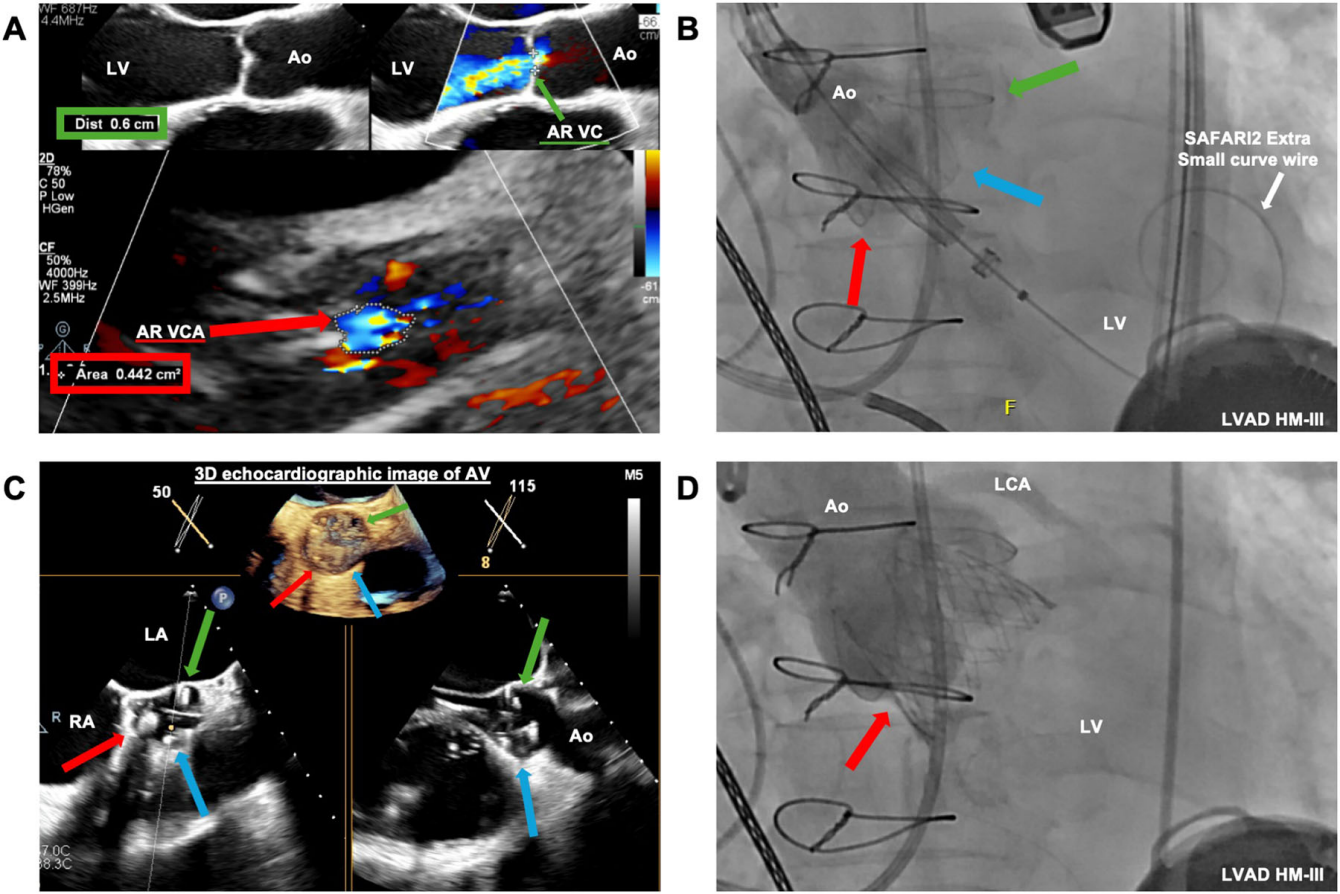
Patient 2. (A) Severe aortic regurgitation with vena contracta (VC) 0.6 cm [red arrow] and VC area (VCA) 0.442 cm2 [green arrow] on transesophageal echocardiography (TEE). (B, C) Claspers of J‐Valve were positioned in aortic sinuses under fluoroscopic and TEE guidance [green arrow—claspers positioned in LCC; blue arrow—claspers positioned in RCC; red arrow—claspers positioned in NCC]. (D) Successful J‐Valve deployment confirmed on fluoroscopy [red arrow—J‐Valve]. [Color figure can be viewed at wileyonlinelibrary.com]

A 55‐year‐old‐man with ischemic cardiomyopathy underwent LVAD implantation with HeartMate III in 2019. He developed symptomatic severe AR (VC 0.6 cm, VC area [VCA] 0.442 cm^2^) with New York Heart Association (NYHA) class IV symptoms in August 2023. Despite optimizing medical treatment with goal‐directed medical therapy including losartan, spironolactone, dapagliflozin, and furosemide, he developed acute decompensated heart failure requiring ICU support in 2023 (Figure 2A). CT analysis revealed tri‐leaflet AV with no calcifications. Aortic annulus diameter, perimeter and area were 2.32 × 2.84 cm, 81.7 mm, and 521.9 mm^2^ respectively on CT (Table 1). Femoral access and coronary heights were favourable for transfemoral TAVI. In anticipation of potential subsequent open sternotomy for heart transplantation as definitive treatment, along with an elevated STS score of 9.46%, the patient was deemed high risk for SAVR. Thus, Heart team consensus was to perform TF‐TAVI with #28 mm J‐Valve under general anaesthesia. As in our first case, warfarin was prescribed peri‐procedurally for LVAD. INR was maintained at 2.0−3.0 peri‐procedurally, with no interruption of warfarin before TF‐TAVI. After crossing the AV with a straight‐tip emerald wire via an AL1 catheter, the J‐Valve was delivered via a 20Fr DrySeal Flex Introducer Sheath (Gore Medical, USA) on a SAFARI2 Extra Small Curve wire (Boston Scientific, USA). The claspers were positioned in the aortic sinuses under fluoroscopic and transesophageal echocardiographic (TEE) guidance (Figure 2B,C). After reducing LVAD speed flow from 5000 to 3000 rpm, the THV was successfully deployed (Figure 2D). LVAD flow speed was subsequently restored to 4000 rpm. ACT was maintained > 300 s with parenteral heparin throughout the procedure. He continued warfarin post‐operatively with INR maintained at 2.0−3.0 for LVAD. Reassessment echocardiography at 6‐month and 1‐year follow‐up showed no residual AR, with no evidence of AV or aortic root thrombus. NT‐proBNP level improved from 1372 to 752 ng/L. He experienced improved NYHA function class, from NYHA IV at baseline to NYHA I post‐operatively. With TF‐TAVI as a bridge to definitive treatment, the patient underwent heart transplantation 17‐months post‐TAVI with clinical recovery.

## 4 Case 3

**Figure 3:**
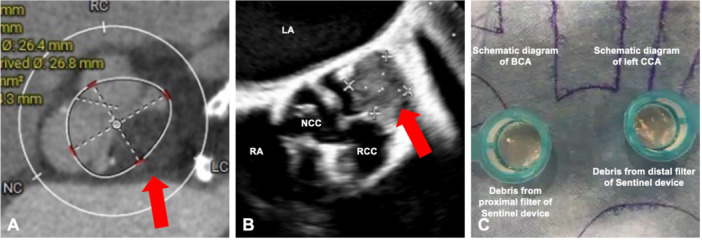
Patient 3. Aortic root thrombus in the LCC [red arrow] was found on (A) computed tomography and (B) transoesophageal echocardiography (TEE). (C) Multiple debris up to 4 × 2mm were captured by SENTINEL embolic protection device. [Color figure can be viewed at wileyonlinelibrary.com]

A 62‐year‐old patient who received CRTD for ischemic cardiomyopathy developed progressive heart failure despite goal‐directed medial therapy, requiring HeartMate III implantation in 2023. By 2024, he had developed symptomatic severe AR (VC 0.7 cm, VCA 0.506 cm^2^) with NYHA Class IV symptoms and repeated hospitalizations for heart failure. Despite goal‐directed medical therapy with sacubitril‐valsartan, spironolactone, and furosemide, he developed decompensated heart failure requiring inotropic support. In view of the acute decompensated heart failure and inotrope‐dependency, the patient was deemed high risk for open SAVR. In consideration of TF‐TAVI, ECG‐gated Cardiac CT for TAVI planning was performed. CT showed aortic annulus diameter 2.50 × 3.10 cm, area 629 mm^2^, and perimeter 90.9 mm (Table 1). A thrombus was incidentally detected in the left coronary cusp (LCC) despite INR level remaining in the therapeutic range (between 2.0 and 3.0) with warfarin throughout (Figure 3A). In anticipation of potential cardioembolic events, the consensus was to perform TF‐TAVI with a #29mm J‐Valve with SENTINEL cerebral embolic protection (Boston Scientific, USA). As per previous cases, warfarin was continued without interruption peri‐procedurally to maintain INR at 2.0−3.0. Intra‐operative TEE confirmed the presence of an LCC thrombus (Figure 3B). After SENTINEL deployment via the right radial artery, the procedure for TF‐TAVI was completed in a similar fashion to our second case. LVAD flow was reduced from 4800 to 3200 rpm for THV deployment, then restored to 4700 rpm. ACT was maintained > 300 s throughout the procedure with parenteral heparin. Multiple particles of debris (up to 4 mm × 2 mm) were captured by SENTINEL (Figure 3C). He continued warfarin post‐operatively with INR maintained at 2.0−3.0 for LVAD. Reassessment echocardiography at 3‐month, 6‐month, and 1‐year follow‐up showed no residual AR, with no evidence of AV or aortic root thrombus. NT‐proBNP level improved from 4814 to 1519 ng/L. He experienced improved NYHA function class, from NYHA IV at baseline to NYHA I post‐operatively.

## 5 Case 4

**Figure 4:**
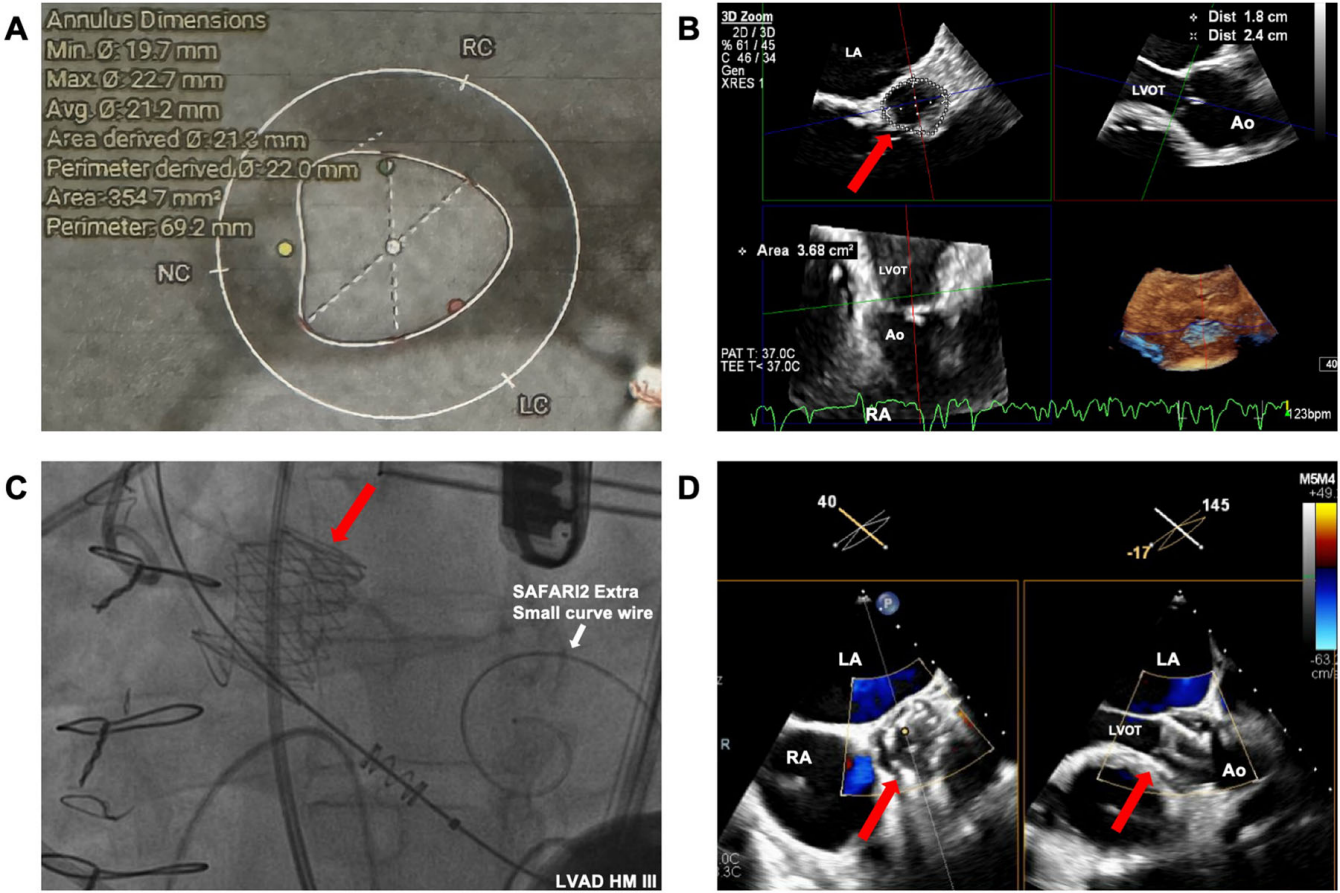
Patient 4. (A) Computed tomography (CT) revealed a small aortic annulus with diameter 1.80 × 2.40 cm, area 368 mm^2^, and perimeter 69.4 mm. (B) Transoesophageal echocardiography (TEE) showing similar measurements [red arrow—aortic annulus]. (C) Successful J‐Valve deployment confirmed on fluoroscopy [red arrow—J‐Valve]. (D) TEE showed no residual AR [red arrow—J‐Valve]. [Color figure can be viewed at wileyonlinelibrary.com]

A 48‐year‐old man with ischemic cardiomyopathy underwent HeartMate III implantation in 2022. He developed symptomatic severe AR (VC 0.8 cm, VCA 0.50 cm^2^) with NYHA Class IV symptoms in 2024. Despite goal‐directed medical therapy, with sacubitril‐valsartan, spironolactone, and furosemide, he remained symptomatic with repeated hospitalizations for heart failure. In anticipation of potential subsequent open sternotomy for heart transplantation as definitive treatment, along with an elevated STS score of 8.49%, the patient was deemed high risk for SAVR. The heart team consensus was to perform TF‐TAVI with J‐Valve under general anaesthesia. CT revealed a small aortic annulus with diameter 1.80 × 2.40 cm, area 368 mm^2^, and perimeter 69.4 mm (Figure 4A). TEE showed similar measurements (Figure 4B) (Table 1). Similar to the previous cases, INR was maintained at a therapeutic level of 2.0−3.0 by continuation of warfarin peri‐procedurally without interruption. TF‐TAVI with a #25 mm J‐Valve was performed using the previously described technique, with ACT maintained > 300 s throughout the procedure with parenteral heparin. LVAD flow speed was reduced from 5000 to 3000 rpm for THV deployment, then restored to 4500 rpm. J‐Valve was successfully deployed with no residual AR at 3‐ and 6‐month echocardiographic assessment (Figure 4C,D). Reassessment echocardiography showed no evidence of AV or aortic root thrombus at 3‐ or 6‐month follow‐up. NT‐proBNP level improved from 15,955 to 4822 ng/L. He experienced improvements in NYHA function class, from NYHA IV at baseline to NYHA I post‐operatively. With TF‐TAVI as a bridge to transplantation, the patient underwent heart transplantation 9 months following TF‐TAVI with clinical recovery.

## 6 Discussion

AR complicates LVAD implantation in up to 25%–30% of recipients [1]. The development of AR in patients with continuous‐flow LVAD is multifactorial and includes commissural fusion and leaflet tissue deterioration [1, 4]. Although the exact mechanism of AV commissural fusion in continuous‐flow LVAD remains unclear, it has been postulated that the absence of antegrade pulsatile flow across the AV results in prolonged leaflet coaptation, promoting commissural fusion and leaflet tethering [5]. Different studies have tried to examine the AV leaflets in continuous‐flow LVAD recipients, with some showing commissural fusion as a consequence of thrombus formation and some showing remodelling of valvular endothelial cells during altered shear stresses [5, 6].

Assessing AR severity in LVAD recipients is challenging. Since AR in these patients exhibits continuous flow, conventional grading parameters may underestimate its severity. Novel echocardiographic measures, such as the ratio of peak velocity during systole to that during diastole of the LVAD outflow cannula and diastolic flow acceleration of the outflow cannula, have shown better correlation with AR regurgitation fraction and clinical filling pressures obtained by cardiac catheterization in patients with moderate to severe AR [7].

The onset of AR in LVAD recipients is often associated with a cascade of hemodynamic sequelae, including right heart failure and malignant arrhythmias [8, 9]. Mortality is significantly higher in these patients, with those experiencing significant AR showing a mortality of 59.5% compared with 37.2% in patients without AR [2]. As such, it may be necessary to perform AV replacement in LVAD patients with severe AR.

Management strategies of AR in LVAD recipients remain challenging. While heart transplantation, a definitive treatment for LVAD recipients with advanced heart failure, should be considered in LVAD recipients complicated with AR, donor heart may not be readily available. Thus, surgical AV intervention has been the conventional treatment for significant AR in LVAD recipients, either as a bridge to heart transplantation or as definitive treatment. Nonetheless re‐operative open heart surgery carries a high risk of complications and mortality in this population, particularly in the context of advanced heart failure with poor left ventricular function aggravated by AR [10]. Furthermore, in anticipation of subsequent re‐sternotomy for heart transplantation surgery as a definitive treatment for this group of patients (as demonstrated in our second case and fourth case), further open heart surgery (e.g., SAVR) poses increased risks for subsequent sternotomy during heart transplantation. Previous studies explored the use of TAVI devices that were not specifically designed to treat AR in this cohort of patients. Nevertheless the use of non‐dedicated devices led to device migration, as seen in our first case [3, 11]. Such THV migration and embolization may also cause interference with the LVAD system [3, 11].

With the advent of dedicated TAVI for pure AR such as the JenaValve system, transfemoral TAVI has reemerged as a promising treatment for AR in LVAD patients [3]. Dedicated systems offer the advantage of mechanisms for active anchoring onto AV leaflets, significantly reducing the risk of device embolization. Use of the transfemoral J‐Valve system for treating AR in an LVAD recipient has been described recently [12]. We report the first case series of consecutive patients with LVAD treated with the transfemoral J‐Valve system for AR, and demonstrate its effectiveness even in challenging anatomies such as the presence of aortic root thrombus. While data regarding long‐term durability of the J‐valve system in LVAD‐recipient is lacking, and newly implanted THVs may be susceptible to accelerated degeneration due to continuous retrograde flow from the LVAD, our case series demonstrated that transfemoral TAVI with the J‐valve system can serve as a potential bridge to heart transplantation.

Several technical considerations are important when performing transfemoral TAVI in LVAD patients. For instance, during AV crossing a pigtail catheter is typically used for patients with pure AR; nonetheless in LVAD recipients whose AVs remain closed throughout the cardiac cycle, a straight tip wire may be more useful. Special attention to wire positioning is essential to avoid entanglement with the LVAD circuit. Moreover, whereas THVs in conventional TAVI tend to migrate toward the aorta, in LVAD recipients the valve may migrate into the ventricle because of the LVAD flow. This necessitates reducing the LVAD speed during deployment and rapid pacing is typically unnecessary. The degree of LVAD flow reduction is critical because an excessively low flow can lead to hemodynamic compromise and clot formation within the LVAD system. In our series, LVAD speed was reduced to approximately 3000 rpm for each case in the setting of HeartMate III LVAD.

Valve size selection is another important consideration [13]. Generally, a CT perimeter‐derived diameter is used to guide THV size selection. The available sizes of TF‐J Valve system range from 22 to 34 mm. Previous cohort studies reported an oversizing rate of −11.5% to 7.53% for transapical J‐Valve [13]. In our case series of LVAD patients with AR, our chosen over‐sizing rate of 1.4%, 7.7%, and 13.6% was safe and resulted in satisfactory AR resolution. Further studies are required to investigate the oversizing strategy when treating pure AR with dedicated TAVR systems.

Peri‐procedural and post‐procedural anti‐thrombotic regimens are of paramount importance in LVAD recipients who are at risk of thromboembolic complications. Most of our patients are prescribed warfarin for anticoagulation, with an INR target of 2.0−3.0. Peri‐procedurally, warfarin was continued without interruption to achieve a therapeutic INR of 2.0−3.0. Intra‐procedural heparin was given to ensure an ACT over 300 s.

As illustrated by our third case, LVAD patients may develop AV thrombus despite adequate anticoagulation. This increases the risk of cardioembolic events during TAVI. Although the routine use of cerebral embolic protection devices remains controversial, their application may be beneficial in LVAD patients with high risk of cardioembolism during the procedure, such as those with a confirmed aortic root thrombus, to minimize the risk of stroke.

## 7 Conclusion

AR complicating LVAD remains a challenging clinical scenario. Dedicated TF‐TAVI systems for pure AR, including the J‐valve system used in our case series, have emerged as effective treatment options for LVAD patients with pure AR, even in the presence of challenging anatomy.

## Conflicts of Interest

Dr. Tang has received speaker's honoraria and served as a physician proctor, consultant, advisory board member, TAVR publications committee member, RESTORE study steering committee member, APOLLO trial screening committee member and IMPACT MR steering committee member for Medtronic, has received speaker's honoraria and served as a physician proctor, consultant, advisory board member and TRILUMINATE trial anatomic eligibility and publications committee member for Abbott Structural Heart, has served as an advisory board member for Boston Scientific, a consultant and physician screening committee member for Shockwave Medical, a consultant for Philips and Edwards Lifesciences, Peija Medical and Shenqi Medical Technology, and has received speaker's honoraria from Siemens Healthineers. The other authors declare no conflicts of interest.
